# Myristoylated, Alanine-rich C-kinase Substrate (MARCKS) regulates Toll-like receptor 4 signaling in macrophages

**DOI:** 10.21203/rs.3.rs-3094036/v1

**Published:** 2023-07-10

**Authors:** Jiraphorn Issara-Amphorn, Virginie Sjoelund, Margery Smelkinson, Sung Hwan Yoon, Nathan P. Manes, Aleksandra Nita-Lazar

**Affiliations:** National Institute of Allergy and Infectious Diseases; National Institute of Allergy and Infectious Diseases; National Institute of Allergy and Infectious Diseases; National Institute of Allergy and Infectious Diseases; National Institute of Allergy and Infectious Diseases; National Institute of Allergy and Infectious Diseases

## Abstract

MARCKS (Myristoylated Alanine-rich C-kinase Substrate) is a membrane protein expressed in many cell types, including macrophages. MARCKS is functionally implicated in cell adhesion, phagocytosis, and inflammation. LPS (lipopolysaccharide) triggers inflammation via TLR4 (Toll-like receptor 4). The presence of MARCKS and the formation of phospho-MARCKS in macrophages have been described, but the role(s) of MARCKS in regulating macrophage functions remain unclear. To investigate the role of MARCKS during inflammation, we activated macrophages using LPS with or without the addition of a PKC inhibitor. We found that PKC inhibition substantially decreased macrophage IL6 and TNF cytokine production. In addition, confocal microscopy revealed that MARCKS and phospho-MARCKS increased localization to endosomes and the Golgi apparatus upon LPS stimulation. CRISPR-CAS9 mediated knockout of MARCKS in macrophages downregulated TNF and IL6 production, suggesting a role for MARCKS in inflammatory responses. Our comprehensive proteomics analysis together with real-time metabolic assays comparing LPS-stimulation of WT and MARCKS knock-out macrophages provided insights into the involvement of MARCKS in specific biological processes and signaling pathways, uncovering specific proteins involved in regulating MARCKS activity upon LPS stimulation. MARCKS appears to be a key regulator of inflammation whose inhibition might be beneficial for therapeutic intervention in inflammatory related diseases.

## Introduction

The myristoylated alanine-rich C kinase substrate (MARCKS) is a ubiquitous and highly conserved membrane protein which is present in many cell types including macrophages^1^. MARCKS is attached to the cell membrane via a myristoylated site at its N-terminus and a cationic AA rich effector domain (ED)^2,3^. The lysine rich ED contains four serine residues which are potential phosphorylation sites. MARCKS was first identified as a protein kinase C (PKC) substrate^4^. PKC phosphorylation or calmodulin-binding on the ED leads to the migration of MARCKS from the cell membrane to the cytosol followed by activation of several signal transduction pathways^5^. MARCKS returns to the cell membrane after dephosphorylation^3,6^. MARCKS is involved in a wide variety of functions such as brain development^7^, phagocytosis^8^, cell migration^9^, cell adhesion and inflammation^10,11^.

Macrophages are innate immune cells that play key roles in inflammation. Pathogen associated molecular patterns (PAMPs) are recognized by pattern recognition receptors (PRRs), which participate in the initiation of specific immune responses^12^. One of the well-known PAMPs is lipopolysaccharide (LPS), a major gram-negative bacterial cell wall component, which is a potent activator of Toll-like receptor 4 (TLR4) of macrophages leading to inflammatory responses. The transcription level of MARCKS is increased in response to LPS stimulation^13^. LPS also induces MARCKS phosphorylation^14^. Moreover, LPS triggers secretion of proinflammatory cytokines such as TNF^15^ and IL6^16^ which contribute to the pathogenicity of many diseases including asthma^17^, rheumatoid arthritis and sepsis^18^. Elevation of TNF and IL6 in the serum are commonly found in those patients^18,19^. The functions of MARCKS in response to the LPS are still controversial. MARCKS was reported to block the effect of LPS stimulation in a study that used MARCKS deficient embryonic fibroblast cells^20^. In contrast, another group reported that MARCKS promotes proinflammatory cytokine expression in macrophages. Inhibition of MARCKS using the myristoylated N-terminal sequence (MANS) peptide suppresses pro-inflammatory cytokines and attenuates sepsis in a mouse model^21^. The discovery of the mechanism by which MARCKS contributes to the inflammatory response may provide new strategies to manipulate inflammation-related diseases.

Our laboratory has shown that MARCKS phosphorylation was upregulated during LPS stimulation using phosphoproteome profiling of immortalized mouse macrophages (IMMs)^22^. In this work, we investigated the effects of MARCKS in the context of macrophage functions in response to LPS stimulation. We have used CRISPR CAS9 technique to create a MARCKS knockout in IMMs and characterized the knockout in comparison to wild type IMMs. We used mass-spectrometry-based proteomics to compare proteome profiles of wildtype and MARCKS deficient macrophages after LPS stimulation to provide a global view of MARCKS-dependent changes in the macrophage proteome. We found that while MARCKS promotes IL6 and TNF production, MARCKS deficient macrophages suppress the OXPHOS (oxidative phosphorylation) pathway to reduce the cytokine production and inhibit the pro-inflammatory functions.

## Results

### MARCKS is upregulated during LPS stimulation

**Figure 1:**
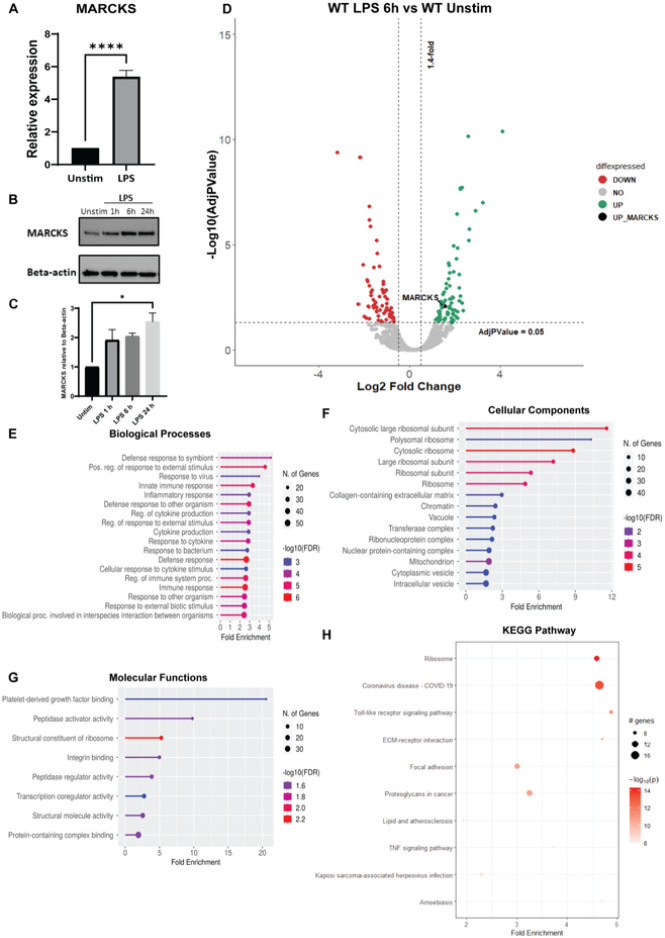
MARCKS is upregulated upon LPS stimulation in macrophages. A) IMMs were treated with 100 ng/mL of LPS for 6 hours. MARCKS mRNA was measured by real-time PCR. Data are representative of 2 independent experiments, shown as mean ± SEM. **** p< 0.0001 (unpaired t test). B) IMMs were stimulated with 100 ng/mL of LPS for 0, 1, 6 or 24 h. MARCKS protein expression was detected by western blot, and beta-actin was used as a loading control. The full-length blot image is included in the Supplementary Information file, Supplementary Figure 3. C) Densitometric analysis of western blot results. MARCKS levels were normalized to beta-actin. Data are representative of 2 independent experiments, shown as mean ± SEM. * p< 0.05 (one-way ANOVA). D) Volcano plot of the differential protein expression within WT IMMs after 6 h LPS stimulation vs unstimulated WT IMMs. Green dots represent up-regulated proteins, red dots represent down-regulated proteins and black dot represents MARCKS. E-G) Go enrichment analysis performed using shinyGO v. 0.77 to characterize the biological functions: D) Biological Processes E) Cellular Components F) Molecular functions of these differentially expressed proteins. H) KEGG pathway analysis of LPS-treated IMMs vs unstimulated IMMs. The x axis represents the fold enrichment value while y axis represents the enriched pathways. The bubble size shows the number of differentially expressed proteins in the indicated pathway. The color intensity indicates the −log10(lowest-p) value, the darker the red, the more significant pathway enrichment.

The functions of MARCKS protein in the context of LPS signaling in macrophages are not fully understood. To examine the relationship between MARCKS protein expression during LPS stimulation, we exposed wild type immortalized mouse macrophages (WT IMMs) to LPS for 6 hours. Then, MARCKS mRNA and protein levels were measured using real-time PCR and western blots. We found that the mRNA of MARCKS increased 5-fold after LPS stimulation (Fig. 1A). The protein expression level of MARCKS also increased after LPS exposure (Fig. 1B).

Mass spectrometry was used to quantitatively compare the proteomes of unstimulated and LPS stimulated WT IMMs. Comparison between LPS-treated and control revealed 270 proteins differentially expressed after LPS treatment (101 down-regulated and 169 up-regulated proteins). As expected, MARCKS was significantly (nearly 3-fold) increased in macrophages exposed to LPS compared to unstimulated control (Fig. 1C). Next, we performed gene ontology enrichment analysis (GO terms) for the up- and down-regulated proteins. The top enriched biological processes highlight GO terms that reflect macrophage functions such as innate immune response, inflammatory response, and cytokine production (Fig. 1D). We further analyzed cellular components, pathways and molecular functions (Fig. 1E, F, G). KEGG pathway analysis indicated enrichment in pathways associated with Toll-like receptor signaling (Fig. 1H). Our data suggest that MARCKS might play a regulatory role in the LPS signaling in macrophages since we found both its mRNA and protein levels change following LPS stimulation.

### MARCKS increases its colocalization with the endosome post LPS stimulation

**Figure 2:**
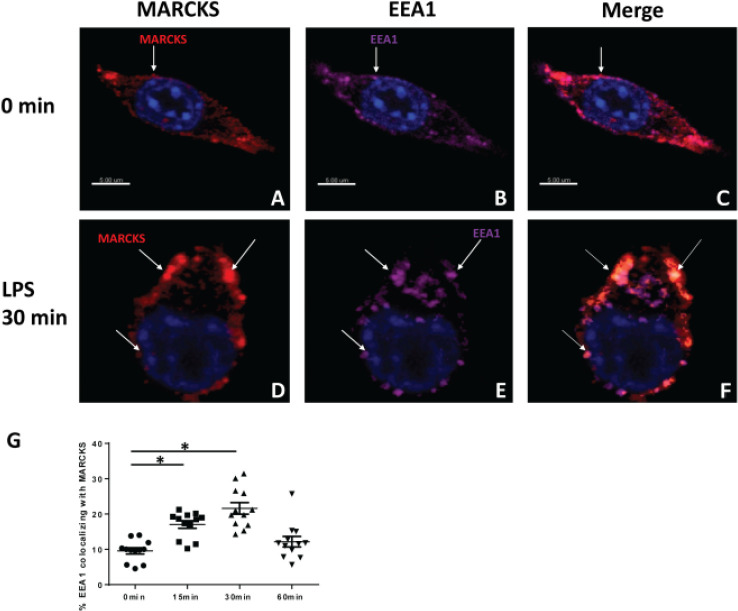
MARCKS increases its localization to the endosome following LPS stimulation. A, D) Immunofluorescent staining of MARCKS (red) in macrophages before and after LPS stimulation. B, E) Immunofluorescence staining of EEA1, the early endosome marker (purple) in macrophages before and after LPS stimulation. C, F) Images merged to show the co-localization of MARCKS and endosome. A-F) The nuclei were stained with Hoechst (blue). G) The percentages of colocalization of MARCKS and EEA1 were calculated. Data are represented as mean ± SEM and *p<0.05 (one-way ANOVA)

Intracellular translocation of TLR4 prevents the constitutive response of cells that are exposed to bacteria and their products. Since both MARCKS and the TLR4 regulator TRAM, that associates with TLR4 and is essential for TLR 4 signal transduction^23^ contain a myristoyl tail, we wanted to investigate whether MARCKS localizes to the same cellular compartments as TLR4 post LPS stimulation in macrophages. We exposed WT IMMs to LPS for 0 15, 30 and 60 minutes. Endogenous localization of MARCKS and EEA1, a marker for early endosomes, were determined by immunofluorescence microscopy. With no stimulation, MARCKS is understood to be tethered at the membrane by its myristoyl tail, and its effector domain is supposed to interact with the membrane. MARCKS was located at the cell membrane prior to LPS stimulation. After LPS stimulation for 30 minutes, MARCKS colocalized with the endosome (Fig. 2A–F) which is in agreement with previous reports^20^. This colocalization increased post LPS stimulation, and returned to basal level 60 min after stimulation (Fig. 2G).

### MARCKS colocalizes with TLR4 at early time points post LPS stimulation

Since MARCKS colocalizes with the endosome post LPS stimulation and it is known that TLR4 is translocating from the membrane to the endosome post LPS stimulation, we wanted to investigate whether MARCKS and TLR4 colocalized and for what amount of time. To answer this question, WT IMMs were stimulated with LPS for 15, 30, or 60 minutes or left unstimulated, and colocalization of endogenous TLR4 and MARCKS was determined with confocal imaging. Based on the Pearson’s coefficient, TLR4 and MARCKS colocalized in the absence of LPS but was disrupted upon stimulation (Supplementary Fig. 1A). However, the amount of MARCKS that colocalized with TLR4 didn’t change significantly during our experimental time, whereas the amount of TLR4 that colocalized with MARCKS decreased during the 60 min interval of the experiment (Supplementary Fig. 1B). Since MARCKS colocalized with TLR4 post LPS stimulation, our results suggest that MARCKS might be physiologically relevant during LPS-activation of TLR4 signaling pathway.

### Phospho-MARCKS localizes to the Golgi post LPS treatment

**Figure 3:**
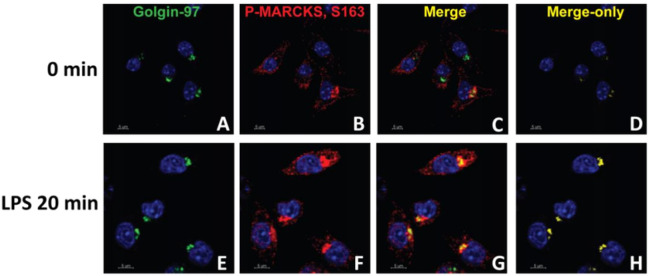
Phospho-MARCKS colocalizes with Golgi following LPS stimulation. A-F) WT IMMs were treated with LPS for 20 min, then Golgin-97, a trans-Golgi network marker (green), phospho-MARCKS (red) and the nuclei (blue) were visualized using specific antibodies. C, G) Images of the same samples merged for analysis of colocalization of MARCKS and the trans-Golgi network. D, H) The merge only panel is shown in yellow.

Numerous studies have shown that upon LPS stimulation, the effector domain of MARCKS is phosphorylated^24,25^, and it is thought that phosphorylation elicits a switch from MARCKS interacting with the plasma membrane to interacting with actin in the cytoplasm. MARCKS phosphorylation on sites Ser152, 156 and 163, or any combinations of these phosphorylated residues^22^ could be contributing to this activity of MARCKS. To investigate the localization of endogenous phospho-MARCKS Ser163, we labeled fixed IMMs with antibodies against phospho-MARCKS Ser163 conjugated to fluorescent secondary antibodies and performed immunofluorescence microscopy. The intensity of phospho-MARCKS in confocal images was very low without LPS treatment but it increased dramatically after LPS treatment. Furthermore, after LPS treatment, the colocalization of phospho-MARCKS with Golgin-97, a trans-Golgi network marker, increased dramatically (Fig. 3).

### MARCKS expression is TLR4-dependent

**Figure 4:**
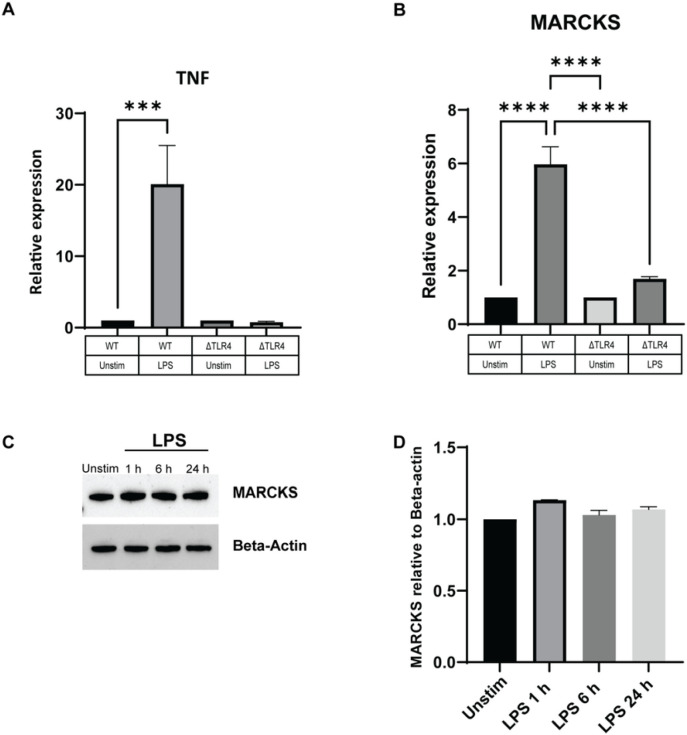
LPS induces MARCKS expression through the TLR4 dependent pathway. A, B) WT IMMs and ΔTLR4 IMMs were treated with LPS for 6 hours or left unstimulated (indicated as “unstim” in this and subsequent panels). TNF and MARCKS mRNA levels were measured using real-time PCR. Data are representative of two independent experiments and shown as mean ± SEM: ***p<0.005, **** p< 0.0001 (one-way ANOVA). C) ΔTLR4 IMMs were treated with LPS for the indicated times. MARCKS protein expression was detected by western blot and beta-actin was used as a loading control. The full-length blot image is included in the Supplementary Information file, Supplementary Figure 3. D) Densitometric analysis of western blot results. MARCKS was normalized to beta-actin. Data are representative of 2 independent experiments, shown as mean ± SEM.

We inquired whether the upregulation of MARCKS post LPS stimulation is dependent on TLR4 signaling. To address this question, we used TLR4 knockout (ΔTLR4) IMMs and WT IMMs and stimulated them with LPS for 0, 1, 6, and 24 hours. ΔTLR4 IMMs failed to increase the TNF mRNA level whereas the WT IMMs showed nearly 20-fold increase in TNF mRNA level after LPS stimulation (Fig. 4A). The level of MARCKS mRNA in ΔTLR4 IMMs was slightly increased but not statistically significant whereas it was significantly increased (~ 6 fold) in WT IMMs treated with LPS (Fig. 4B). Of note, MARCKS protein level did not change after LPS stimulation in TLR4 knockout IMMs (Fig. 4C, D). This is in contrast to the LPS-induced increase in MARCKS protein observed in WT IMMs (Fig. 1B), suggesting that MARCKS mRNA expression and protein abundance are TLR4 dependent.

### MARCKS promotes proinflammatory cytokine responses

**Figure 5:**
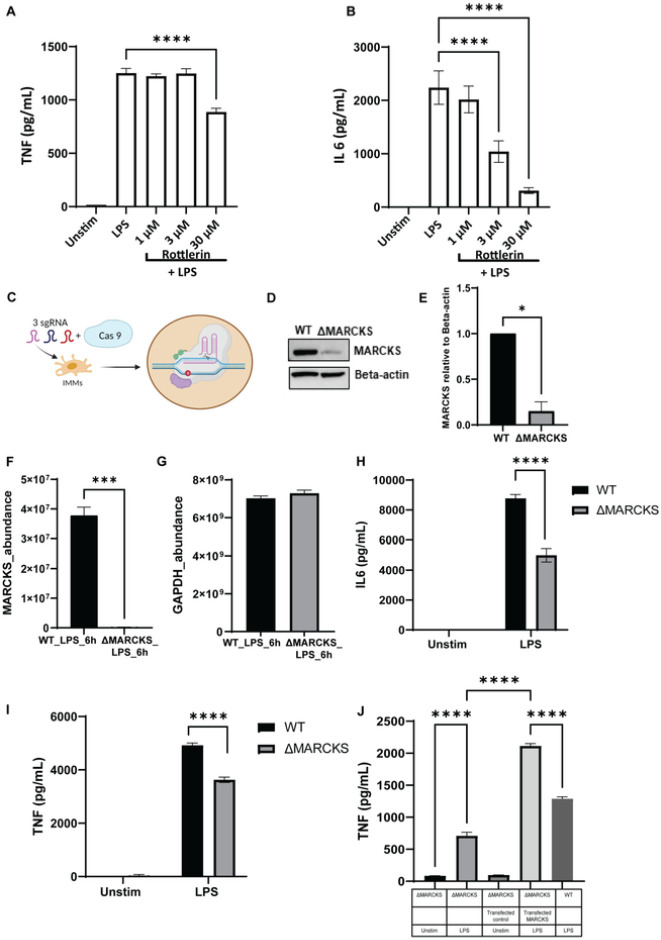
MARCKS deficient macrophages downregulate pro-inflammatory cytokine production. A, B) WT IMMs were pre-treated with PKC inhibitor rottlerin for 1 hours, and then treated with LPS for 6 h or left unstimulated (indicated as “unstim” in this and subsequent panels). TNF and IL6 were measured by ELISA. Data are representative of two independent experiments and shown as mean ± SEM: **** p< 0.0001 (one-way ANOVA). C) The schematic of the experimental strategy for CRISPR CAS9 mediated MARCKS knockout in macrophages. D) Cell lysates of WT and ΔMARCKS IMMs were used analyzed by western blot to compare MARCKS protein expression level and beta-actin was used as a loading control. The full-length blot image is included in the Supplementary Information file, Supplementary Figure 3. E) Densitometric analysis of western blot results. MARCKS was normalized to beta-actin. Data are representative of 2 independent experiments, shown as mean ± SEM. * p< 0.05 (unpaired t test). F, G) Comparison of the MARCKS protein abundance between WT and ΔMARCKS IMMs obtained from mass spectrometry (F). GAPDH was used as a housekeeping protein control (G). The sum of the LC-MS peptide peak area was used as a measure of relative protein abundance. H, I) WT and ΔMARCKS IMMs were treated with LPS (100 ng/mL) for 24 hours, and IL6 (H) or TNF (I) was measured by ELISA. Data are representative of 2 independent experiments and shown as mean ± SEM: **** p< 0.0001 (two-way ANOVA). J) WT, ΔMARCKS IMMs and ΔMARCKS knock-in IMMs were treated with LPS (100 ng/mL) for 24 hours, TNF was measured by ELISA. Data are representative of 2 independent experiments and shown as men ± SEM: **** p< 0.0001 (one-way ANOVA).

Macrophages play a pivotal role in the inflammatory response, but the link between MARCKS and macrophage-mediated inflammation remains to be elucidated. During LPS treatment, MARCKS is thought to be phosphorylated by PKC. To examine whether MARCKS has an effect on the inflammatory responses, we pre-incubated IMMs with rottlerin, a PKC inhibitor, for one hour before stimulation with LPS and compared them to cells that did not receive rottlerin. We found that PKC inhibition attenuated the proinflammatory cytokine secretion of both TNF and IL6 (Fig. 5A, B). Next, we used CRISPR CAS9 mediated gene knockout to generate MARCKS knockout in IMMs (ΔMARCKS IMMs). The schematic diagram for generating the knockout cells is shown in Fig. 5C. The level of MARCKS protein expression was confirmed using western blot, which showed nearly no MARCKS protein in the ΔMARCKS IMMs when compared to WT IMMs (Fig. 5D, E). To further confirm the knockout was successful, we used mass spectrometry to compare the levels of MARCKS protein between WT IMMs and ΔMARCKS IMMs. The results showed that there is no protein detected in ΔMARCKS IMMs while WT IMMs showed 3.6×10^7^ protein abundance (Fig. 5F). Of note, both WT and ΔMARCKS IMMs had comparable levels of glyceraldehyde-3-phosphate dehydrogenase (GAPDH), a housekeeping protein we used as a control (Fig. 5G). Moreover, looking at the tryptic peptides identified by mass spectrometry, we detected no MARCKS peptides in ΔMARCKS IMMs whereas in WT IMMs we identified 6 peptides from MARCKS. (Supplementary table 1). These data confirmed that we successfully created MARCKS deficient macrophages.

To confirm the role of MARCKS in the pro-inflammatory cytokine responses, we exposed WT and ΔMARCKS IMMs to LPS, then and collected the conditioned media for measurements of cytokine production. We found that ΔMARCKS IMMs downregulated both TNF and IL6 levels. Our data suggest that MARCKS knockout suppressed LPS-induced IL6 and TNF expression (Fig. 5H, I). To further confirm the role of MARCKS in the cytokine production, we cloned MARCKS sequence to pLenti-C-mGFP-P2A-Puro, a lentiviral vector with C-terminal mGFP tag and P2A-Puromycin and then we transduced it into ΔMARCKS IMMs to generate stable cells (MARCKS knock-in IMMs). In the stable cell line, we activated MARCKS knock-in IMMs with LPS and compared them to WT and ΔMARCKS IMMs. MARCKS knock-in IMMs rescued the levels of TNF cytokine production (Fig. 5J). These data are in agreement with the PKC inhibitor and LPS stimulated MARCKS knockout macrophage data. The results further suggest that MARCKS promotes TNF and IL6 production in macrophages post LPS stimulation.

### MARCKS deficient macrophages show distinct proteomics profile during LPS stimulation

**Figure 6:**
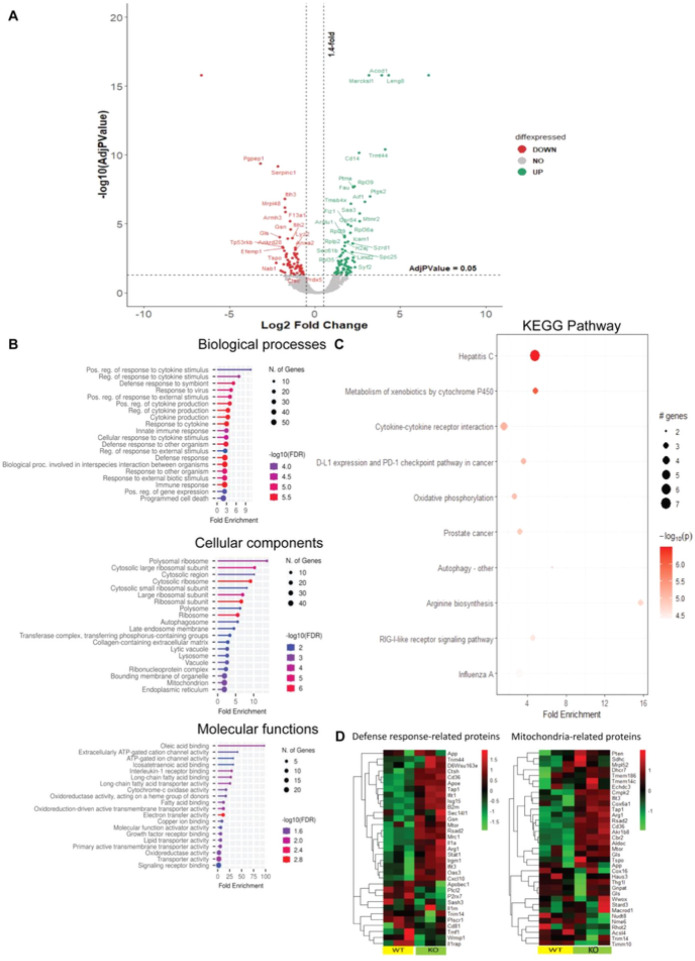
MARCKS-deficient macrophages show distinct proteome profiles after LPS stimulation. A) Volcano plot for the differential protein expression after 6h LPS stimulation between WT IMMs and ΔMARCKS IMMs. Green dots are representative of up-regulated proteins and red dots are representative of down-regulated proteins. B) Go enrichment analysis was performed using shinyGO v 0.77 to characterize the biological functions including Biological Processes, Cellular Components and Molecular Functions of these differentially expressed proteins. C) KEGG pathway enrichment analysis. The x axis represents the fold enrichment value while y axis represents the enrich pathway the size of bubble shows the number of differentially expressed proteins in the indicate pathway. The color indicates the -log10(lowest-p) value, the redder color, the more significantly pathway is enriched. D) Heatmap plot of differentially expressed proteins involved in defense response related proteins and mitochondria related proteins comparing WT and ΔMARCKS IMMs.

Because MARCKS is also associated with a wide variety of functions in cells such as signal transduction, cell migration, cell proliferation, cell differentiation and cytokine production, we hypothesized that phenotypic changes in ΔMARCKS macrophages may be associated with other proteins that regulate macrophage functions. To test this hypothesis, we stimulated WT and ΔMARCKS IMMs with the LPS for 6 hours and conducted a proteomic analysis using label free quantification. We found 94 up-regulated proteins and 60 down-regulated proteins in ΔMARCKS after LPS treatment compared to WT IMMs after LPS treatment (Fig. 6A). The identified proteins were primarily associated with biological processes involved in the macrophage functions such as “Positive regulation of response to cytokine stimulus”, “Regulation of response to the cytokine stimulus” and “Innate immune response”. The enriched cellular components represented “Polysomal ribosome” and “Mitochondrion”. The molecular functions included “ATP gate channel activity”, “Electron transfer activity” and “Cytochrome C oxidase activity”. Taken together, these data suggested that mitochondria might be involved in the phenotypic changes in IMMs upon LPS stimulation in the absence of MARCKS. Since we found several GO terms of the top 15 cellular components and molecular functions were associated with mitochondrial proteins (Fig. 6B), we performed KEGG pathway enrichment analysis of the significant proteins in ΔMARCKS IMMs with LPS treatment compared to WT IMMs. Significantly enriched pathways included “Oxidative phosphorylation” and other metabolic pathways including “Metabolism of xenobiotics by cytochrome P450” and “Cytokine-cytokine receptor interaction” (Fig. 6C). We created a heatmap of the protein abundance in individual samples based on ontology enrichment analysis of the significant proteins focusing on innate defense response-related proteins and mitochondria related proteins. We found that the protein expression patterns in the individual samples of WT and ΔMARCKS IMMs after LPS treatment were clearly distinct (Fig. 6D), with many proteins showing an increase in KO cells. Our proteomics data suggest that the classic mitochondria function such as oxidative phosphorylation (OXPHOS) might be altered during LPS signaling in ΔMARCKS IMMs.

### MARCKS promotes oxidative phosphorylation

**Figure 7:**
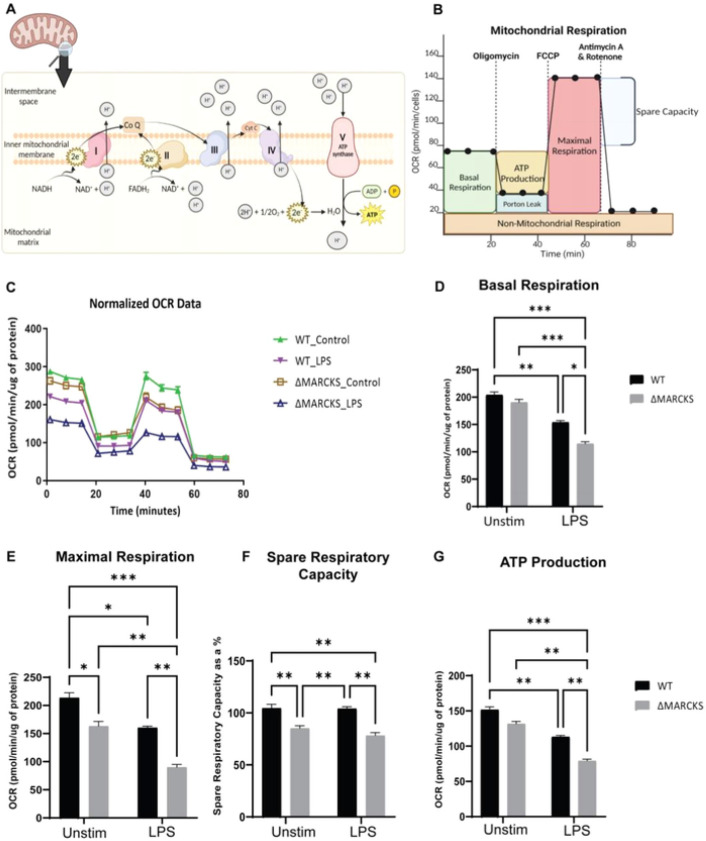
ΔMARCKS IMMs are deficient in mitochondrial respiration. A) Illustration of oxidative phosphorylation in the mitochondrial inner membrane. B) The general pattern of a mitochondrial stress test. C) WT and ΔMARCKS IMMs were treated with LPS (100 ng/mL) for 6 hours. Oxygen consumption rates (OCR) were measured using Seahorse with indicated inhibitors. The data were normalized to total protein. D-G) The individual parameters for basal respiration (D), maximal respiration (E), spare respiratory capacity (F) and the ATP production (G) were obtained from the Wave software program. WT and ΔMARCKS IMMs were treated with LPS (100 ng/mL) for 6 hours or left unstimulated (indicated as “unstim” in this and subsequent panels). Data are representative of 2 independent experiments and shown as mean ± SEM: *** p< 0.005 **p<0.01 * p<0.05 (two-way ANOVA). All the statistically significant differences are indicated.

Mitochondrial OXPHOS is the electron transfer process through the main 5 protein complexes in the inner mitochondrial membrane resulting in the production of adenosine triphosphate (ATP), the main source of energy in eukaryotic cells^26^. The schematic diagram for the OXPHOS and the oxygen consumption rate over time is shown in Fig. 7A, B. To directly test if MARCKS influences OXPHOS, we used extracellular flux analysis to measure OXPHOS during LPS treatment of WT and ΔMARCKS IMMs compared to unstimulated control. In the untreated control group, we found that ΔMARCKS IMMs had lower OXPHOS when compared to the WT IMMs. After 6 h LPS treatment, the mitochondrial respiration was downregulated in both WT and ΔMARCKS IMMs. (Fig. 7C). We further analyzed the other parameters obtained from extracellular flux analysis. We did not observe any significant difference in basal respiration between unstimulated WT and ΔMARCKS IMMs (Fig. 7D), but when the cells were stimulated with LPS, basal respiration statistically decreased in ΔMARCKS cells compared to the WT IMMs. Maximal respiration and spare respiration were significantly decreased in ΔMARCKS IMMs when compared to WT IMMs. In the LPS treatment group, LPS reduced the maximal respiration in both cell types but that of ΔMARCKS IMMs was still lower compared to WT IMMs (Fig. 7E, F)

Mitochondria play a crucial role in ATP production, and mitochondrial dysfunction may result in insufficient energy supply for the cells. Moreover, ATP is required during the immune response^27,28^. We aimed to determine whether ATP production is defective in ΔMARCKS IMMs, resulting in lower proinflammatory cytokine production. To address this, we examined the ATP production in OXPHOS during mitochondrial respiration. We found that ATP production in ΔMARCKS IMMs was reduced when compared to WT IMMs. ATP production was significantly lower after LPS treatment (Fig. 7G). In addition, LPS-treated-ΔMARCKS IMMs showed statistically significant lower ATP production than the WT IMMs. Our data suggest that MARCKS is involved in mitochondrial respiration, resulting in sufficient energy production for macrophage inflammatory functions such as cytokine production.

## Discussion

The macrophage response to the LPS resulting in the cytokine production involves many elements of regulation such as downstream signaling transduction, transcriptional regulation, regulation by adaptor proteins^29^, and subcellular localization. Our results showed that both MARCKS mRNA and protein levels (as detected by both western blots and proteomics) were upregulated after LPS stimulation. In addition, LPS induced changes of the macrophage proteome, and MARCKS was among the upregulated proteins. A previous report showed that MARCKS transcription level is highly elevated after LPS stimulation^13^, but the mechanisms of action remained unclear. Our findings provide evidence that MARCKS plays a key role during LPS stimulation in macrophages and significantly contributes to the inflammatory response.

TLR4 is translocated to the endosome after LPS stimulation^30^ and many pieces of indirect evidence suggest that MARCKS is physically connected to the TLR4 signaling pathway. Considering the well-characterized function of the TIR-domain containing adaptor protein TRAM, which is a transmembrane protein containing the myristoylated site and PKC phosphorylation site^31^, which are both important for innate immune signaling, and the colocalization of TRAM with TLR4 in the plasma membrane and Golgi apparatus where the TLR4 signal transduction takes place^23,32^, we sought to find out whether translocation of MARCKS protein from the cell membrane to cytoplasm may have a similar effect. We observed that MARCKS co-localized with TLR4 and endosome at the early time points following LPS stimulation. In addition, phosphoS163-MARCKS also co-localized with the Golgi apparatus which is the same cellular compartment where TLR4 translocates eventually after LPS stimulation. Colocalization of MARCKS and endosome agrees with the previous report by Mancek-Keber et al.^20^ However, in that study, the cytokine production after LPS stimulation was attenuated by MARCKS and MARCKS effector peptide in cultured cell lines and mouse embryonic fibroblasts^20^. This result was surprising and is in contrast to two more recent studies which showed that MARCKS enhanced proinflammatory cytokine expression by regulation of p38/JNK and NF-kappaB, an effect which was further inhibited by MARCKS inhibitory peptide (MANS) in neutrophils^33^ and macrophages and a mouse model^10^. Additionally, inhibition of MARCKS using MANS peptide resulted in a reduction of proinflammatory cytokines such as CXCL1, IL-1β, IL6, MCP-1 and TNF in bronchoalveolar lavage fluid in a neutrophil elastase–induced murine bronchitis model^34^

We sought to address this controversy, and our results using IMMs demonstrated that the increased expression of MARCKS protein is functionally associated with elevated pro-inflammatory cytokine production and that MARCKS deficient macrophages had significantly reduced levels of secreted IL6 and TNF. Furthermore, inhibiting MARCKS activation with a PKC inhibitor reduced cytokine production. Therefore, our results strongly favor the hypothesis that MARCKS is indeed a positive regulator of inflammatory function in macrophages.

MARCKS knockout mice are not well-characterized because MARCKS deficiency in mice resulted in abnormal brain development and the pups die within several hours after birth^7^. Therefore, we used instead the CRISPR CAS9 technique^35^ for the first time to generate MARCKS knockout macrophages and confirm that cytokine production is altered during LPS stimulation. These results further confirmed that MARCKS promotes IL6 and TNF expression during LPS stimulation in macrophages. Because inflammatory cytokine secretion by macrophages is a major cause of pathogenesis and progression of the inflammatory related diseases, a better understanding of the regulatory mechanisms of cytokine production would be beneficial for the identification of therapeutic targets. Because MARCKS is expressed in many innate immune cells including macrophages^13^, neutrophils^36,37^ and monocytes^38^, which are the key player cells for the inflammatory responses, inhibition of MARCKS should lead to a reduction of the proinflammatory cytokines produced by many innate immune cell types.

In addition, we report for the first time the global changes in proteome profiles of MARCKS deficient macrophages compared to WT IMMs. Interferon-induced protein with tetratrico-peptide repeats-1(IFIT1) and interferon-induced protein with tetratrico-peptide repeats-3 (IFIT3), both of which play an important role during viral infection, are upregulated in ΔMARCKS IMMs. IFITs play an important role during viral infection^39^. In contrast, IFIT1 and IFIT3 have been identified as negative regulators of LPS-induced TNF in human macrophages^40^. Upregulation of IFIT1 and IFIT3 in our proteomic data may be the cause of the lower cytokine production phenotype in ΔMARCKS IMMs. Gene ontology analysis and KEGG pathway enrichment analyses of the up- and down-regulated proteins pointed to the oxidative phosphorylation. Oxidative phosphorylation occurs mainly in the mitochondrial inner membrane. Glycolysis and mitochondria are the main sources of energy production during inflammatory responses^41,42^. Cellular energy production changes, and ATP dependent cytokine production involved in inflammation have been described^43,44^. Our data indicate that ΔMARCKS IMMs have decreased mitochondrial respiration compared to the WT IMMs. A previous study showed that more mitochondria or an increase in glycolytic respiration resulted in higher ATP production which supported macrophage cytokine production^45^. Moreover, mitochondrial respiration is lower in the LPS-tolerant macrophages^46^. Our results point to the correlation between MARCKS, mitochondrial respiration and cytokine production.

**Figure 8:**
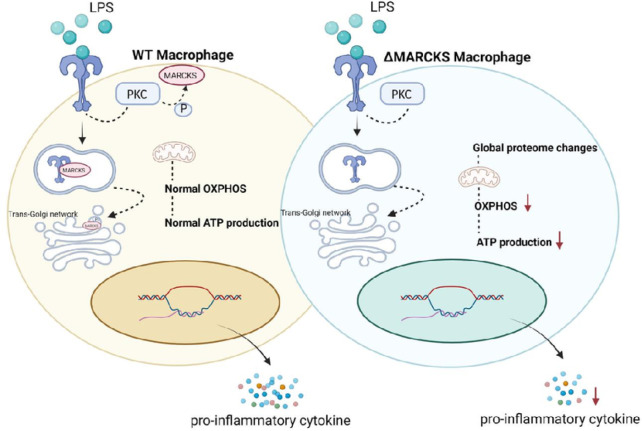
Hypothetical mechanism explaining the effect of LPS signaling in MARCKS deficient macrophages. In response to the LPS signaling in macrophages, LPS binds to the TLR4 and the signaling cascade is initiated. In WT macrophages after LPS stimulation, PKC is activated and MARCKS is phosphorylated. Then, MARCKS migrates from the plasma membrane to the cytoplasm and can colocalize with TLR4 at an early endosome. Phospho-MARCKS also co-localizes with the trans-Golgi network where the TLR4 signal transduction is taking place resulting in the pro-inflammatory cytokine production. In contrast, MARCKS deficient macrophages induce global proteome changes resulting in decreased OXPHOS and the ATP production. The reduction of OXPHOS and the ATP production results in low energy status of the macrophages which may cause the lower cytokine secretion.

In conclusion, MARCKS promotes proinflammatory cytokine production during LPS stimulation in part by increasing the cell’s ATP production. The hypothesis we propose is shown in Fig. 8. The direct interactions between MARCKS and other inflammatory pathway proteins remain unknown, and the precise mechanisms of action need further investigation. MARCKS appears to be an emerging therapeutic target in inflammatory diseases.

## Materials and Methods

### Cell line

Immortalized mouse macrophages (IMM) (a generous gift from Dr. Eicke Latz ^47,48^) were cultured in complete DMEM (Gibco, Grand Island, NY, USA) complemented with 10% FBS (Gibco), in a 5% CO_2_ incubator at 37°C, maintained at low densities and passaged until reaching the confluent state, usually every 3–4 days on sterile tissue culture plates.

### Real-time PCR

The RNeasy kit (QIAGEN, Hilden, Germany) was used for isolation of total RNA from cell culture. Total RNA (1ug) was converted to cDNA using MultiScribe reverse transcriptase (Thermo Scientific, Rockford, IL, USA), and real-time PCR was performed using *Power* SYBR Green PCR Master Mix with specific primer to determine gene expression level. The primers are listed in supplementary Table 2. The ^ΔΔCt^ method was used for determining relative gene expression and beta-actin was used as a housekeeping gene.

### MARCKS cloning

Mouse MARCKS was amplified by PCR using primer containing 15 bp overlap complementary to the vector ends at the 5’ end of the forward and reverse primers (5’-AGATCTGCCGCCGCGATCGCATGGGTGCCCAGTTCTCC-3’) and (5’-GCGGCCGCGTACGCGTTTACTCGGCCGTTGGCGC-3’). The PCR products were cloned into third-generation lentiviral vector, pLenti-C-mGFP (OriGene, Rockville, MD, USA) using infusion enzyme (TAKARA, Kusatsu, Japan). Plasmid sequences were verified by Sanger sequencing at Psomagen Inc (Rockville, MD, USA).

### Stable expression by lentiviral transduction

HEK293FT cells (Thermo Scientific) were seeded on 6 wells cell culture plates (250,000 cells/well) overnight prior to transfection. pLenti-C-mGFP with MARCKS sequence were transfected to HEK293FT cells via TranIT-TKO (MirusBio, Madison, WI, USA). Conditioned media containing lentiviral particles were harvested 24h and 48h post-transfection and filtered using 0.45 μM filters (Millipore, Carrigtwohill, Ireland) before being used to infect IMMs cells. Cells were monitored under fluorescence microscopy. Stable cell transfectants were selected using puromycin (5 μg/mL) (Gibco) containing media. Single cells were isolated and cultured for 10–14 days to obtain homogenous clonal populations. MARCKS expression levels were confirmed using western blots.

### CRISPR CAS 9-mediated MARCKS gene knockout

CRISPR Cas9-mediated MARCKS knockout was generated by Cas9 ribonucleoprotein using the Neon Transfection System Starter Pack (Invitrogen) according to the manufacturer’s instruction. Briefly, three single guide RNA (sgRNAs) that contain MARCKS targeting sequences and a Cas9 nuclease-recruiting enzyme were designed using CRISPR-Cas9 guide RNA design checker (www.idtdna.com/CRISPR-Cas9). IMMs were collected and washed once with PBS. Then, 4×10^6^ cells were electroporate with Cas9 protein v.3 (Integrated DNA technologies, San Diego, CA, USA), complexed with 3 sgRNAs (seed sequences: 5’-CACGTCGTCGCCCAAGGCGG-3, 5’-TGGCCACGTAAAAGTGAACG-3’, 5’-AGCAAGAAGGAGTCGGGCGA-3’) in the nucleofector buffer (Invitrogen, Waltham, MA, USA) and cultured for 3–4 days. The knockout candidates were screened using western blot and mass spectrometry.

### Western blot analysis

IMMs were lysed in Pierce RIPA buffer (Thermo Fisher Scientific) supplemented with Halt protease inhibitor (Thermo Fisher Scientific) and phosphatase inhibitor cocktails (Thermo Fisher Scientific). Total protein concentration was determined by BCA protein assay (Thermo Fisher Scientific). Samples (20 μg total protein) were loaded into NuPAGE 4 to 12% Bis-Tris polyacrylamide gels (Invitrogen, Carlsbad, CA, USA) and run at 200 V for 1 h. The proteins were transferred to the PVDF membrane (Thermo Fisher Scientific). The membranes were blocked in 5% BSA overnight and then incubated with specific primary antibodies for one hour followed by incubation with secondary antibodies for one hour. The antibodies are listed in supplementary Table 3. The blots were developed using ECL substrate (Thermo Fisher Scientific) The results were visualized using a ChemiDoc imaging system (Bio-Rad, Hercules, CA, USA). The densitometric analysis of western blot results was performed using Image J.

### ELISA

TNF and IL6 were quantified using ELISA kits (Invitrogen, Vienna, Austria) kit according to the manufacturer’s instructions. Briefly, IMMs were treated with 100 ng/mL LPS (LPS from *Salmonella minnesota* R595, Enzo Life Sciences) for 6 hours in quadruplicate and the supernatants were collected at the indicated time points. Capture antibody was applied to 96 well plates overnight followed by blocking with 1x diluent buffer for one hour at room temperature, then supernatants with appropriate dilution factor were put in each well for two hours at room temperature. Detection antibody and streptavidin-HRP were sequentially added to the assay plate and incubated one hour and 30 minutes respectively. ELISA washing steps were performed 4 times with 0.05% tween 20 (Thermo Fisher Scientific) in PBS (PBS-T). ELISAs were developed with TMB substrate for 10 mins followed by a stop solution. Absorbances were determined using a microplate reader. The results were interpreted in comparison to the standard curve.

### Seahorse Assay

IMMs in different experimental groups (control vs LPS 6 hours) were dispersed into monolayers for the measurement. Mitochondrial stress tests were performed at 37°C using the Seahorse XFe96 bioanalyzer (Seahorse Bioscience). IMMs were seeded at 2× 10^5^ cells per well on the Seahorse analysis plates. Oxygen consumption rates (OCR) and extracellular acidification rates (ECAR) for the mitochondria were measured in XF media (containing 25 mM glucose, 2 mM L-glutamine, and 1 mM sodium pyruvate) under basal conditions and in response to 1.5 μM oligomycin, 1 μM fluoro-carbonyl cyanide phenylhydrazone (FCCP), and 0.5 μM rotenone and antimycin A. All of the values were normalized with total protein using Wave software (Agilent).

### Mass spectrometry

#### Sample preparation.

WT and MARCKS knockout IMMs were seeded on a 10 cm dish and cultured overnight, the cells were unstimulated or stimulated with 100 ng/mL LPS for 6 hours. The indicated samples were lysed using RIPA buffer (Thermo Fisher Scientific). For each sample, 500 ug of total protein mass was run on Bis-Tris NuPAGE gel (Invitrogen) as described above. The gels were fixed using fixing solution (47.5% methanol and 5% glacial acetic acid) for 30 minutes at room temperature. The fixed gels were stained using PageBlue^®^ protein staining solution (Thermo Fisher Scientific) for one hour at room temperature, and then destained overnight with ddH_2_O at 4°C. Each lane was cut into 1 mm^3^ pieces using razor blades and the gel pieces were put in 1.5 mL Eppendorf tubes and processed using in-gel digestion according to the published protocol and summarized below^49^.

#### In-gel protein digestion:

Briefly, 500 μL of acetonitrile (ACN) were added to the gel pieces and incubated for 10 minutes at room temperature before removing all the supernatant from the tube. For reduction of disulfide bond, 50 μL of DTT in 100 mM ammonium bicarbonate (ABC) was added to the tube and incubated at 56 ° C for 30 minutes. Then, 50 μL of 55 mM of 2-chloroacetamide (CA) in 100 mM ABC solution were added and incubated for 20 minutes at room temperature in the dark. Then, trypsin solution (Promega, Madison, WI, USA) (1:25 w:w ratio) was added and the samples were incubated at 37°C for 17 hours. Following incubation, peptides were cleaned and desalted using C18 ZipTip tips (Millipore) according to the manufacturer’s instruction.

#### Mass Spectrometry.

An Orbitrap Fusion Eclipse with an EASY-Spray ion source (Thermo Fisher Scientific, San Jose, USA) coupled to a Thermo UltiMate 3000 (Thermo Fisher Scientific) was used for LC-MS/MS experiments. 1 ug of total peptides were injected for LC-MS/MS analysis. Peptides were trapped on an Acclaim C18 PepMap 100 trap column (5 μm particles, 100 Å pores, 300 μm i.d. × 5 mm, Thermo Fisher Scientific) and separated on a PepMap RSLC C18 column (2 μm particles, 100 Å pores, 75 μm i.d. × 50 cm, Thermo Fisher Scientific) at 40 C. The LC steps were: 98% mobile phase A (0.1% v/v formic acid in H_2_O) and 2% mobile phase B (0.1% v/v formic acid in ACN) from 0 to 5 min, 2–35% linear gradient of mobile phase B from 5 to 155 min, 35–85% linear gradient of mobile phase B from 155 to 157 min, 85% mobile phase B from 157 to 170 min, 85–2% linear gradient of mobile phase B from 170 to at 172 min, 2% of mobile phase B from 172 to 190 min. Eluted peptides were ionized in positive ion polarity at a 2.1 kV of spraying voltage. MS^1^ full scans were recorded in the range of m/z 375 to 1,500 with a resolution of 120,000 at 200 m/z using the Orbitrap mass analyzer. Automatic gain control and maximum injection time were set to standard and auto, respectively. Top 3sec data dependent acquisition mode was used to maximize the number of MS^2^ spectra from each duty cycle. Higher-energy collision-induced dissociation (HCD) was used to fragment selected precursor ions with normalized collision energy of 27. MS^2^ scans were recorded using an automatic scan range with a resolution of 15,000 at 200 m/z using the Orbitrap mass analyzer. Data analysis, label free quantification and statistical analysis were performed using Proteome Discoverer 2.5 (Thermo Fisher Scientific). Briefly, the raw files were searched against the mouse uniport database with the list of common protein contaminants. The groups and conditions were specified to obtain the quantification ratios and adjusted p-values were calculated using Benjamini-Hochberg method. The data visualization was performed using R packages. The differentially expressed proteins were identified using log2 fold change less than − 0.5 (0.7-fold change) for downregulated proteins and log2 fold change more than 0.5 (1.4-fold change) for upregulated proteins combined with the adjusted p-value less than 0.05. Volcano plots were generated by ggplot2. All the significant proteins were used as input to identify the significant enriched Go and pathway analysis. KEGG pathway analyses were search against mouse KEGG genome database and the bubble chart was generated by pathfindR. The adjusted p-value were calculated using Bonferroni method. The heatmap was generated by pheatmap. Go enrichment analysis was performed using shiny v. 0.77 by searching against the mouse string database with the FDR cutoff 0.05. The mass spectrometry proteomics data have been deposited to the ProteomeXchange Consortium^50^ via the PRIDE^51^ partner repository with the dataset identifier PXD042097 (temporary reviewer account details: **Username**: reviewer_pxd042097@ebi.ac.uk **Password**: cyYoee0O).

### Confocal microscopy

IMMs were seeded in 24 well dishes with an inserted glass coverslip at 2×10^4^ cells per well and incubated overnight to recover. The cells were then treated with 1 mg/mL LPS for 5, 10, 15, 20, 30, 45, 60 minutes or left untreated for the phospho-MARCKS colocalization study, 15, 30, 60 minutes and untreated for the endosome colocalization study and 1, 5 minutes and untreated for Golgi colocalization study. After LPS treatment, the cells were washed with ice cold PBS three times and fixed with 2% paraformaldehyde for 20 minutes at room temperature. The coverslips were washed three times with PBS-T, the primary antibody was added in PBS-T with 0.1% BSA and incubated overnight at 4°C. After extensive washing with PBS-T, the secondary antibody was added and incubated in the dark on the coverslips for 1 hour at room temperature. The coverslips were washed once with PBST, Hoechst dye was added (1:15000) and incubated in the dark for 15min after which the coverslips were again extensively washed with PBST. The coverslips were mounted on slides using ProLong Gold (Life Technologies, Grand Island, NY) and kept in the dark at 4°C until visualization. The visualization of the image was performed using confocal Imaging was performed using a Leica SP8 confocal microscope and data quantification including colocalization analysis was performed using Imaris software.

### Statistical Analyses

Statistical analyses were performed using Prism 9.0 (GraphPad software, Inc., La Jolla, CA, USA).
